# QRS Voltage Changes in Heart Failure: A 3-Compartment Mechanistic Model and its Implications 

**Published:** 2010-10-31

**Authors:** John E Madias

**Affiliations:** Mount Sinai School of Medicine of the New York University, and the Division of Cardiology, Elmhurst Hospital Center, New York, NY

**Keywords:** Heart failure, electrocardiogram, peripheral edema, mechanistic heart models, QRS amplitude attenuation

## Abstract

A 3-compartment mechanistic model is proposed to explain the attenuation of the electrocardiographic QRS complexes (↓QRSV) observed in patients with heart failure (HF). This includes the effects of increased intracardiac blood volume and decreased hematocrit due to blood dilution (1st compartment), the heart's alteration in electrogenesis due to possible ischemia or inflammation, leading to myocardial edema, (2nd compartment), and the passive volume conductor of the tissue and organ constituents of the thorax and the entire body, with their resistivity changes due to increased fluid content (pulmonary and peripheral edema) (3rd compartment). The clinical implications of the model are outlined.

## Background

**Figure 1:**
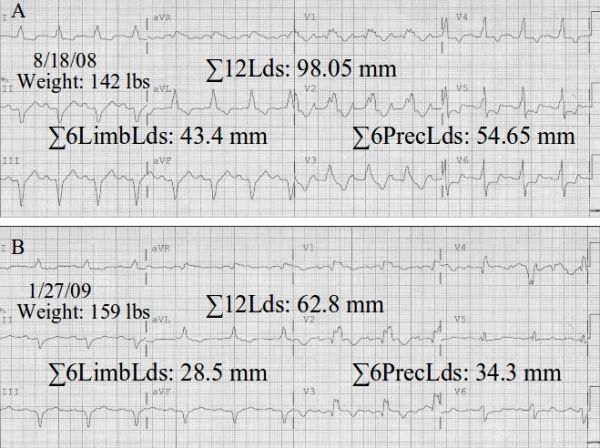
ECGs A and B were recorded using Philips Page Writer touch electrocardiographs, at a speed of 25 mm/sec, calibration of 10.0 mm = 1.0 mV, and frequency response of 0.05 - 150 Hz. cutoffs. Measurements of peak-to-peak amplitudes of the QRS complexes were obtained from the commercially available for routine use automated morphology algorithm of the Philips Trace MasterVue Management System; sums of the 6 limb leads (ΣLimbLds),  6 precordial leads (ΣPrecLds),  and all 12 ECG leads (Σ12Lds), were manually calculated.

Figure 1 depicts attenuation of the electrocardiographic (ECG) QRS complexes (↓QRSV) in a 53 year old male patient with a New York class III stage C nonischemic cardiomyopathy and a left ventricular ejection fraction of <20%. He had suffered decompensation of his heart failure (HF), associated with worsening of his symptoms, peripheral edema, and an increase of 11.97% of his body weight. This weight gain was associated with a 34.33% decrease in the sum of peak-to-peak amplitudes of the QRS complexes from the 6 limb leads, with corresponding decreases of 37.24%, and 35.95%, of the sums of the 6 precordial leads, and all 12 ECG leads. The patient did not have rales on lung auscultation, had jugular venous distension and complained of dyspepsia which was attributed to resultant gastrointestinal venous congestion. The inverse is also true, i.e., augmentation of the QRS complexes (↑QRSV) observed in association with loss of weight in patients with HF responding to diuresis. These perturbations in the amplitude of QRS complexes and body weights are apparent by reviewing data of patients with HF followed over long time periods. The repeatability in the same patient and the reversibility in the process suggest that this QRS complex amplitude/weight relationship is a real phenomenon. Although this ECG correlate of HF was noted more than 50 years ago, its mechanism has been elusive,  [1] and its appreciation and employment among physicians continues to be poor. An attempt to explain the mechanism via its attribution to the Brody effect  [2], has led to a contradiction since according to this theoretical construct, cardiomegaly (increased intracavitary blood mass) often associated with HF is expected to produce ↑QRSV, the opposite of what is actually observed in the ECG with clinical decompensation  [1]. The Brody effect deals with the distorting impact of the body's inhomogeneities on the registered ECG and it is attributed to the difference in electrical conductivity of the blood mass and the heart itself plus the entire body volume conductor. While the conductivity of the heart and the lungs is comparable, the one of the intracavitary blood mass is ten times greater. Consequently the intracavitary blood mass is expected to exert a short-circuiting effect on the potentials generated by the heart. Although this was initiated as an idealization, it has been confirmed when applied to different heart models. Also the concept has been shown to be correct in experimental studies, but controversy has arisen often, particularly when authors invoke the Brody effect in a simplistic big heart volume, large QRS complexes conceptualization. It should be emphasized in the outset that as per Brody effect what is enhanced with the increase in the intracavitary volume and/or conductivity  are the radially conducted electrical vectors, while the tangentially conducted electrical forces are attenuated  [2]. However following diuresis and management of HF ↑QRSV is detected in the ECG, instead of the expected ↓QRSV, as per Brody effect  [1]. In contrast this author for sometime has attributed such perturbations of the ECG and body weight in patients with HF exclusively to the influence of peripheral edema,  [3] not unlike what happens to patients with volume overload from a variety of causes, but not HF  [4].   In all these systemic edematous states, the increased fluid content, and in turn decreased resistivity, or increased conductivity, of the passive body volume conductor surrounding the heart, leads to reduced recorded surface potentials, as the electrical current emanating from the epicardial surface is transferred to the recording sites  [4].

However the issue may be more complex, as this author has realized on scrutinizing the figures of a long ago published animal study  [5]. In that work, ↓QRSV in intracavitary and pericardial electrograms (EGMs), was recorded in a canine model of HF with elevated (left ventricular diastolic pressures) (LVDP), produced by restricting coronary flow, or by increasing the venous inflow. Unfortunately no surface ECGs were recorded in that study, to provide information on what is recorded at the body surface. Combining the experience from this experimental study,  [5] and observations in patients with HF,  [3] one may conclude that HF leads to ↓QRSV in both intracavitary, pericardial electrograms (EGM), and surface ECGs. Moreover, some experience of this author in recording EGMs via a saline-filled indwelling central venous catheter (quasi-intracardiac EGM) in patients with edematous states but not HF, where the surface ECG showed ↑QRSV while the quasi-intracardiac EGM remained unaltered following veno-venous ultrafilltration with removal of large amount of fluids,  [4] leads to the conclusion that ↓QRSV in both intracardiac EGMs and surface ECGs is a feature of HF, in contrast to other non-HF related edematous states where only the surface ECG is affected  [4].  This differentiation between HF and other edematous states imply that although both share in the emergence of peripheral edema, the former may be a more complex state in which the heart itself, plays a role in causing ↓QRSV. Attempting to attribute solely the changes of QRS complex amplitudes to Brody effect or peripheral edema implies a 1-compartment model, which may not be realistic.

**Figure 2:**
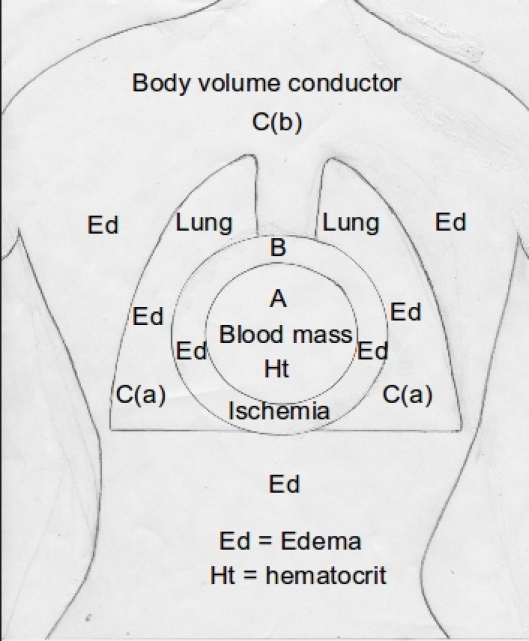
A 3-compartment model of HF, showing compartment A (intracardiac), which can be influenced by changes in the intracavitary blood mass and the hematocrit, compartment B (the heart), which can be influenced by edema and ischemia, and compartment C (body volume conductor), which includes the lungs [C(a)] and the rest of the body [C(b)], which can be influenced by edema; the separation of the lungs and the rest of the body is an analogy introduced to accommodate the clinical states of lung congestion [C(a)] and/or peripheral edema [C(b)].

A 2-compartment model, considers the heart and the enveloping volume conductor, and although it proposes a specific mechanism for the latter compartment, via which it impacts the recorded ECG, it does not do so for the former  [6]. Based on the previously cited animal work,  [5] myocardial ischemia/injury due to coronary flow deprivation or volume overload, both resulting in persistently elevated LVDP led to stable ↓QRSV in the intracavitary EGMs (no influence herein of the surrounding volume conductor), but via what mechanism? How does the heart itself, when in failure, impacts the ECG?  When contemplating this, one should consider not only the volume expansion (dilatation of cardiomegaly) and the elevated LVDP, but also the histological and electrogenetic status of the failing myocardium. Indeed additional influences on the ECG and intracardiac EGMs need to be considered, such as the controversial Brody effect, i.e., the role of dilatation of failing heart,  [2] the decreased hematoctit (Ht) due to the HF induced hemodilution,  [7,8] and possible changes of the cardiac generator itself during HF. Accordingly a 3-compartment model appears necessary to encompass all plausible influences of HF on the ECG, consisting of a) the intracavitary blood mass/volume with its consistency, b) the heart itself, and c) the passive total body volume conductor (Figure 2)

## First compartment

The increased intracavitary blood mass resulting from cardiac dilatation, clinically appreciated by imaging as cardiomegaly, accompanying decompensation in patients with HF, is expected to produce ↑QRSV, while clinical improvement following diuresis with decrease in the heart size is expected to lead to ↓QRSV, according to the Brody effect.  However the opposite to the above, i.e., ↓QRSV and ↑QRSV, respectively, is actually found in patients with HF suffering decompensation, or successfully managed, which has caused the puzzlement of the authors of a previous study who attempted to invoke the Brody effect in explaining the association of reduction of heart size and ↓QRSV in their patients, admitted with decompensated HF and who responded to therapy  [1]. Rudy and Plonsy have attributed this contradiction of what the Brody effect postulates  [2] and the clinical evidence,   [1] to the confounding effect of pulmonary congestion, i.e., ↑QRSV  due to cardiomegaly is counteracted and indeed overwhelmed by the ↑QRSV, effected by the attenuating influence of congested lungs enveloping the dilated heart  [9]. These authors provide a plausible resolution of the controversy employing a mathematical model and appear to favor the notion that the modulating factor reversing what is expected from Brody effect is the high lung conductivity due to congestion, although they also refer to a possible change in the configuration and/or strength of the cardiac field [9]. The modulating effect of the lungs is not borne out by this author's experience,  [10] who has followed in the ambulatory setting patients with HF and fluctuating peripheral edema and body weights but without lung congestion by physical examination, who have shown major changes in the QRS amplitudes with good correlation with their changes in body weights, always contradicting the expectations according to the Brody effect.  However this experience has limitations, since it does not include objective documentation of changing heart sizes or fluid content of the lungs. Thus it is conceivable that patients with HF and no evidence of lung congestion by clinical evaluation (while they manifest evidence of peripheral edema) have increased lung fluid content. The role of the change in the configuration and/or strength of the cardiac field has not been determined, but it is conceivable that one of the 2 or both may be influential in HF and resulting cardiomegaly  [9]. Thus it is possible that weakening of the strength of the cardiac field and its configuration (radial vs tangential activation of the heart) to a different degree, and for different time points and duration during the ventricular activation, and for different parts of the left and right ventricle set the stage for alterations in the QRS amplitude which follow, after all, the postulate of the Brody effect, even if it appears that the results are contradictory. In thinking about this, one should remember that the Brody effect postulates that an increase in the intracavitary blood mass enhances the vectors from the radial spread of excitation and attenuates the vectors from tangential activation  [2]. Indeed Rudy and Plonsey, commenting on this  [9] have attributed the contradictory results of Nelson et al to the fact that these authors deployed their artificial current dipole inside the dogs' heart in their experiments and in an improper fashion, i.e., tangentially, rather than radially, which is what really happens during the normal endocardium to epicardium activation. An additional issue that these 2 authors tackle is the theoretical possibility that the impact of the increase in the intracavitary blood volume on the surface ECG potentials, may be further enhanced by considering that the area of double layer electrical source on the heart is not constant, but it expands with cardiac dilatation  [9]. Thus, it is conceivable that the Brody effect with all its consequences is applicable to the heart volume/QRS amplitude relationship more than it is apparent, in experiments or clinical applications, when implemented comprehensively.

Another matter always neglected is the role of Ht changes during the perturbations of HF course. It has been shown that Ht decreases during cardiac decompensation in patients with HF (hemodilution), and increases on recovery (hemoconcentration), with a negative correlation of the changes in the body weight and Ht noted  [7]. Indeed changes in Ht could be employed to estimate body fluid status in patients with HF  [7]. This observation has mechanistic connotations along the Brody effect postulate, according to which the enhancement of the radial cardiac forces is based on the lower resistivity of the blood mass in comparison with the heart muscle or the surrounding tissues, and since blood resistivity is directly related to the Ht, changes in the latter are expected to affect the transmission of the cardiac currents to the surface of the body, producing alterations of the QRS voltage  [8].   Accordingly patients with thalassemia who had ECGs before and after transfusions of concentrated red blood cells (to avoid increasing the intracardiac blood volume, a confounding factor herein), showed decreases in the ECG R-waves due to the resulting rise in the Ht which in turn reduced the blood/cardiac tissue difference of resistivity, as per Brody effect  [8]. The authors of this article also commented on the fact that patients with an elevated Ht tend in general to have ECGs with low QRS voltage  [8].

## Second compartment

As stated above the increase in the intracardiac blood mass may have an effect on the strength of the electrical source dipole,  [9] either increasing it as the ventricular surface increases, or decreasing it as the ventricular surface decreases. Also total or partial, generalized or regional alterations in the pattern of ventricular activation in patients with HF (from radial to tangential) may impact the surface ECG potentials, via the Brody effect viewed beyond the volume expansion effect (1st compartment) and via alterations on the heart itself (2nd compartment) (Figure 2). There is no experimental evidence that electrogenesis (action potentials and velocity of conduction) is altered during onset, progression or amelioration of HF. The authors of the canine study cited above  [5]. noted certain ECG changes as HF developed and progressed consisting sequentially of ST-segment elevation in the intracavitary lead, ST-segment depressions and T-wave inversion and subsequent ST-segment elevation in the epicardial leads with a decrease in QS complexes of the intracardiac EGMs to initially 50% and then 25% of their baseline. Finally there were positive T-waves in the epicardial leads and decrease in the amplitudes of the QRS complexes in the EGMs of the epicardial leads. While the above changes appeared in HF due to curtailment of coronary flow, there was no ST-elevation in the intracavitary EGM when HF resulted from an increase in the venous inflow. The ECG patterns observed by these authors were reminiscent of subendocardial ischemic injury progressing eventually to involve the subepicardial heart layer. These changes in the EGMs and ECGs correlated with the changes in LVDP. Also such changes were transient and when coronary flow was restored or increase in the venous inflow was terminated the EMGs and ECGs returned to normal although hemodynamic abnormalities persisted for long periods of time in the animals who had HF imparted by coronary flow deprivation  [5]. This study addresses specifically the changes in the ECG imparted by alterations in the heart itself; however the confounding factor of Brody effect cannot be ascertained in this study since there were no measurements of changes in the heart dimensions. However if cardiac dilatation had occurred, it would have been expected to impart a ↑QRSV, the opposite of what was recorded. Certainly the modulating influence of peripheral volume conductor had been eliminated in this study which implemented the open chest-close pericardium model  [5]. Although patients with HF often show changes in ST-segment and T-waves in their ECG, such changes are attributed to other underlying pathologies like myocardial ischemia, old myocardial infarction, ventricular hypertrophy, conduction abnormalities, or electrolyte disturbances. It would be of interest to systematically evaluate in patients whether there exist ST-segment and T-wave abnormalities, similar to the ones described above, which they could be specifically attributed to HF and its course. It is conceivable that such ST/T changes in humans with HF are subtle because they represent global involvement of the heart with significant cancellation. Also the spreading of a possible initially subendocardial ischemic injury, inherent to HF, toward the epicardium may lead to an amelioration of the severity of the ST-segment depression, conversion to an isoelectric ST-segment, and then ST-segment elevation with an upright T-wave as seen in the animal model, [5]. with this later stage taken as benign in the clinical setting, perhaps e.g., signifying early repolarization. In contrast to such changes, genuine ischemia or ischemic injury, imparted by a regional cause of pathology is expected to result in significant ST-segment depression and elevation. But what was the mechanism of the ST/T wave changes observed in the canine HF model, particularly when they occurred with an increased volume inflow rather than with reduction of coronary flow? Were they related to induced ischemia or were they due to myocardial stretch imparted by cardiac distension? The relevant literature does not refer to ST/T-wave changes but merely to the QRS complex amplitude changes with myocardial stretch. However the changes in the QRS amplitude noted were the ones expected via the mechanism of Brody effect (1st compartment) and not because of influences of stretch on the heart muscle itself (2nd compartment) (Figure 2). This was shown in an isolated rabbit heart modified Langendorff preparation, where changes in the volume and LVDP had an inverse relationship with the amplitude and the slope of unipolar epicardial EGMs when the intracavitary changes were imparted by ionically permeable balloons, but no changes in the EGMs when changes in volume and LVDP were engendered by ionically impermeable balloons, attesting to the role of the increased conductive intraventricular volume, and not to myocardial stretch or wall thickness, in causing the changes in the EGMs  [11]. It should be understood herein that the reduction in the amplitude of the EGMs was not in violation of the Brody effect, since the placement of the electrode by the investigators for recording and ventricular pacing at a basal epicardial site in this experiment records and elicits respectively tangentially conducted ventricular excitation (vide supra)  [11].

Reference has been made in the past by this author only to the effects of the extracardiac organs and tissues (3rd compartment) (Figure 2) when confronted with ↓QRSV in patients with HF  [3]. However one wonders whether this is in error, and here is why. The heart, along with the other organs and tissues is expected to partake in the pathophysiological derangements occurring in the setting of HF. Thus pericardial effusion is present along with lung congestion, pleural effusion, and peripheral edema to a different degree in individual patients, and they are part and parcel of the pathophysiologic expression of HF. The high LVDP and right atrial pressure noted in patients with HF must have the same effect on the atrial and ventricular cardiac tissues (myocardial edema), as in all the other body tissues (lung tissue edema and peripheral edema), since the distribution of sodium and water in the myocardial tissue (extracellular and eventually intracellular) is determined by the same Starling hydrostatic and oncotic forces operating in all other body tissues and organs  [12]. Marked increase in the sodium, chloride and water content per gram of myocardial tissue was detected in a canine model of chronic HF, and felt to be concentrated in the extracellular space  [13]. Myocardial interstitial edema results from increase in coronary venous pressure consequent to right atrial pressure, has been studied in acute and chronic canine models,  [14]. and is a common feature of many medical and surgical conditions  [15,16].  The increased interstitial fluid content of myocardial edema consequent to HF must have a significant effect on the ventricular conduction velocity and is expected to cause ↓QRSV. Myocardial edema results in decrease in resistivity (special resistance of myocardium with normal body fluid concentration is 83-130 Ω-cm, that of body fluid is 64.0-65.2 Ω-cm),  [17] which enhances electrical conductivity of the fluid engorged tissue. It has been long suspected that this is the case, and that edema of the interstitial myocardium and the myocardial intravascular space leads to ↓QRSV  [18]. However elegant work employing an isolated, arterially perfused rabbit papillary muscle model  [19]. showed that the ventricular conduction velocity, and in turn the amplitude of the extracellular EGM, are partially determined by the conductivity of the extracellular compartment, which consists of the interstitial space and the microvascular space. By altering selectively the conductivities of the interstitial space and the microvascular compartment the authors showed that only the former mediates extracellular conductivity, and that the latter is electrically insulated from the interstitial space. Increased water content of myocardial tissue leads to decrease in the amplitude of the recorded intracavitary and epicardial EGMs, and by extension surface ECGs. This was most probably the reason that the ventricular conduction increased and the recorded EGMs decreased when the authors of a highly quoted work  [20]. compared in situ canine hearts with the same hearts perfused with Tyrode solution in a Langendorff apparatus, something that they could not explain at the time.

There is vast literature supporting the view that HF is characterized by an active inflammatory state with cytokines and other inflammatory mediators playing a role in its progression  [21]. In this respect HF shares with myocarditis some pathogenetic links leading to myocyte loss, inflammation, remodeling, and myocardial edema. It is not surprising that many cases of myocarditis lead to an acute or occasionally chronic HF. Myocardial edema has been documented in myocarditis, and has caused a compelling reversible concentric hypertrophy due to fluid engorgement  [22]. Thus, could the reversible ↓QRSV in a case of myocarditis, attributed by this author solely to peripheral edema,   [23]  have been partially due to reversible myocardial edema?  There is an intense activity in the literature looking for myocardial edema (tissue characterization) in myocarditis and many other conditions employing magnetic resonance imaging,  [24,25]. and strain Doppler echocardiography  [26]. Using such modalities with the ECG in tandem could lead to the definition of a role for serial ECGs in the diagnosis, monitoring, and follow-up of patients with HF. In the same vein some aiuthors recently speculated that the ↓QRSV seen in patients with primary hypothyroidism and pericardial effusion may be partially due to myocardial edema, with the heart sharing the increased tissue fluid as the pericardial sack (pericardial effusions), pleural spaces (pleural effusions)and the entire body (peripheral edema) [27,28].

## Third compartment

This has been referred to above, and it has been the only component to which this author has attributed so far the ↓QRSV encountered in patients with HF  [3]. A qualification is in order herein: while the body volume conductor, a passive element in the transfer of electrical currents from the heart to the recording sites at the body surface is conceptually viewed in toto, in influencing the potentials in the limb leads, its thoracic component primarily may be considered as impacting the precordial leads. This was clearly shown in recumbent patients in the Coronary Care Unit with non-HF peripheral edema with impressive fluid sequestration in the dorsal and medial planes of the body, which led to equally impressive ↓QRSV in leads V4-V6  [4]. Another matter of importance is the modulating effect of pulmonary congestion (not always present in patients with HF) which definitely impacts the precordial leads, and may influence to a lesser degree the limb leads, by causing further ↓QRSV, due to an additional layer of low resistivity, interspersed between the heart and the ECG recording sites. This is important as a confounding element in the assessment of ↓QRSV in patients with HF, since clinical presentations of HF with peripheral edema include different degrees of lung congestion from clear lungs to florid pulmonary edema. Mechanistically the impact of lung congestion on the amplitude of the ECG surface potentials should be viewed in a similar context as the influence of pericardial effusion, hydromediastinum, pleural effusion, and bronchopulmonary lavage,  [29] all of which produce ↓QRSV  [30]. The later was showed conclusively to produce ↓QRSV via the imparted reduced lung resistivity in isolation (3rd compartment), in the absence of changes in heart volumes (1st compartment) or associated heart pathology (2nd compartment) (Figure 2).

## Clinical implications

The ECG is serially obtained in patients hospitalized with HF and in those followed in an ambulatory setting. Mere eye-balling, manual or automated measurements of the peak to peak amplitudes of the QRS complexes in serial ECGs reveal a relationship of ↓QRSV and peripheral edema and/or weight gain. Indeed because of the lag in the clinical detection of peripheral edema,  [31] and the notorious unreliability of patient weight measurements, employing the ECG a physician could quickly train oneself to estimate a patient's edematous state and how close or far to his/her dry weight point has been. Gaining weight in patients with HF does not imply always peripheral edema development, and the ECG could act as an arbiter in resolving whether the excess weight is due to peripheral edema or not. Lung congestion can precipitate further transient ↓QRSV, to what was present due to peripheral edema, particularly involving the precordial ECG leads. The intracavitary EGMs are markedly attenuated in HF and such changes could impact sensing or determination of the heart rate in rate adaptive algorithms of pacemakers, intracardiac mapping of areas of ischemia and infarction, sites of slow conduction proned to reentrant arrhythmias, and cardiac rejection in patients with cardiac transplants  [11]. Finally the above conceptualizations (Figure 2) may induce the still untapped potential uses of device-based EGMs in the diagnosis, follow-up and management of patients with HF.
